# Effect of Omega-3 on Recurrent Aphthous Stomatitis and Improvement Quality of Life

**DOI:** 10.1155/2021/6617575

**Published:** 2021-02-10

**Authors:** Zahra Hadian, Ali Akbar Moghadamnia, Sohrab Kazemi, Atena Shirzad

**Affiliations:** ^1^Student Research Committee, Babol University of Medical Sciences, Babol, Iran; ^2^Pharmacology & Toxicology, Neuroscience Research Center, Health Research Institute, Faculty of Medicine, Babol University of Medical Sciences, Babol, Iran; ^3^Pharmaceutics Sciences, Neuroscience Research Center, Health Research Institute, Faculty of Medicine, Babol University of Medical Sciences, Babol, Iran; ^4^Oral Medicine, Oral Health Research Center, School of Dentistry, Babol University of Medical Sciences, Babol, Iran

## Abstract

**Objective:**

Recurrent aphthous stomatitis is one of the most common chronic inflammatory diseases in oral mucosa. Beneficial effects of omega-3 supplements on some inflammatory diseases have been proved. The aim of present study was to evaluate the effect of omega-3 supplements in recurrent aphthous stomatitis management and improve oral health-related quality of life.

**Methods:**

In this double-blind clinical trial, 40 patients with minor recurrent aphthous stomatitis were randomly divided into case and control groups. The case group received 1000 mg capsules of omega-3, while the control group received placebo capsules for 6 months. The questionnaires of the ulcer severity score and the chronic oral mucosal disease questionnaire were filled by the patients in three steps, at the baseline session, after 3 months, and after 6 months. The data were analyzed by SPSS 22 software through ANOVA, Mann–Whitney, and chi-square tests. *P* < 0.05 was considered as significant.

**Results:**

In the omega-3 group, the ulcer severity score showed significant reduction by three-month and six-month follow-ups (*P* < 0.0001, *P* < 0.0001, respectively). The mean score of the chronic oral mucosal disease questionnaire significantly improved by three-month and six-month follow-ups in the omega-3-receiving group.

**Conclusion:**

Use of omega-3 oral supplements decreased the severity of aphthous ulcer and improved oral health-related quality of life.

## 1. Introduction

Recurrent aphthous stomatitis (RAS) is one of the most common chronic inflammatory disorders in the oral cavity affecting about 5–25% of the population. The disease is categorized into three clinical variants including minor, major, and herpetiform aphthous, the most common form of which is minor RAS [1–3]. Aphthous ulcers are self-limiting; however, severe pain and recurrence of lesions in the oral mucosa cause difficulties in oral functions, such as eating, talking, and generally reduces the quality of life in patients [1, 3–5].

The RAS is a multifactorial disorder; thus, studies suggest several factors involved in its etiology. Immunological disorders, hematological deficiencies, stress, trauma, smoking, food allergy, and endocrine changes are among these factors [3, 4]. Recently, many studies have focused on the association between immunological factors and RAS [6]. Considering the diversity in pathogenesis of RAS, several therapeutic methods are discussed; however, in most cases, the treatment is only symptomatic [2]. The goals of recent therapies are to control pain, improve functional limitations, and reduce the ulcer duration and its recurrence [3, 4]. To achieve these objectives, various topical and systemic medications (corticosteroids, anti-inflammatory medications, immunomodulatory agents, and antiseptic agents) have been used [7–10].

The beneficial effects of omega-3 oral supplements on some chronic inflammatory diseases such as rheumatoid arthritis, systemic lupus erythematosus, chronic periodontitis, and the inflammatory bowel disease have been proved [11–17].

Omega-3 polyunsaturated fatty acids have a potential anti-inflammatory effect and are inexpensive, safe, and accessible dietary supplement. Studies have indicated the effect of omega-3 polyunsaturated fatty acids on the reduction of inflammatory biomarkers, cytokines, eicosanoids, and CRPs [18–21]. In addition, eicosapentaenoic acid (EPA) and docosahexaenoic acid (DHA) are the two members of the omega-3 fatty acid family having anti-inflammatory and immunomodulatory characteristics [22]. Some studies have recommended a diet rich in about 1.5 g/d of EPA/DHA to control inflammatory processes [23].

Few studies have investigated the effects of omega-3 on the management of RAS. Therefore, the aim of this study was to evaluate the effect of omega-3 oral supplements on recurrent aphthous ulcers and oral health-related quality of life.

## 2. Methods

### 2.1. Study Design and Ethics

This is a double-blind clinical trial which was registered in the Ethics Committee of Babol University of Medical Sciences with the code of MUBABOL REC.1396.67 and the Iranian Registry of Clinical Trials with the code of IRCT20160112025986N2.

### 2.2. Sample Size and Data Collection

Forty individuals with recurrent aphthous stomatitis referring to Babol Faculty of Dentistry were included in the study. They were randomly divided into two groups: 20 patients in each. The case group received omega-3 capsule while the control group consumed the placebo capsule. All patients were informed of the objectives of the study and written consent was obtained from them.

Inclusion criteria for this study included the following: age over 18, having recurrent aphthous ulcer with at least one ulcer per month, presenting 1–3 minor aphthous ulcers that have occurred at least 48 hours ago, no anesthesia and paresthesia, and usual diet for seafood (one or twice a week). The exclusion criteria were any known systemic disease, pregnancy and lactation, aphthous-like ulcers indicative of systemic diseases such as ulcerative colitis or Behçet's syndrome, severe anemia, use of medications such as systemic corticosteroids, immunomodulator agents, antibiotics, nonsteroidal anti-inflammatory agents (except for occasional use for headache) in the last one month, use of any type of supplements and vitamins in the last one month, and use of topical medications in the last two weeks.

Patient selection was made by an oral medicine specialist. Then, the demographic data of patients, the questionnaire of the ulcer severity score (USS), and the COMDQ questionnaire were filled by the patients.

Considering the double blindness of the study, the patients and the medication distributor did not have any information on the medications used. The case group received omega-3 capsules (1000 mg) prepared by 120 g of DHA and 180 g of EPA provided by Zahravi Company. The control group, on the other hand, received placebo capsules similar to the omega-3 capsules which contained flour. Both capsules had the same labels. Then, the pillboxes were given one of the two codes. The patients were randomly divided into two groups. A group of patients received code 25BC50 and the other received 20AC54 code.

### 2.3. COMDQ and USS Questionnaires

The COMDQ questionnaire is an evaluation tool for the quality of life of patients with chronic mucosal disease which consists of 26 items in four sections: (a) pain and functional limitation (9 items); (b) medication and treatment (7 items); (c) social and emotional (6 items); and (d) patient support (4 items). For every item, there is a 5-point Likert scale from “No, never” (point 0) to “Most often” (point 4). The sum of all scores ranges from 0 to 104 where the results are analyzed as the higher the score, the lower the quality of life [24]. This questionnaire was translated into Persian by Shirzad et al. and its validity and reliability were verified [25].

In the RAS severity questionnaire, patients have been recording the characteristics of the ulcers for the last three months. These characteristics included the average number of ulcers, average duration of ulcers, ulcer frequency period, mucosal site, and VAS criteria for pain. Thus, from these factors, a USS criterion was obtained which determined the severity of the disease [26, 27].

The method of medication use (three times a day for 6 months) was described for the patients. Two follow-ups, 3-month and 6-month sessions, were considered for the patients, and the questionnaires were completed again by them in every session.

### 2.4. Statistical Analysis

After collecting data, they were analyzed by SPSS 22 software via ANOVA, Mann–Whitney, and chi-square tests. *P* < 0.05 was considered to be significant.

## 3. Results

**Figure 1:**
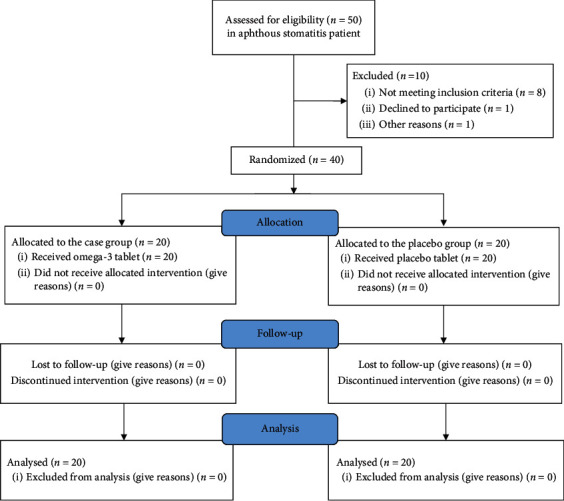
Study flowchart.

From 50 patients with RAS, 10 patients were excluded. 40 remaining patients were divided into two groups randomly. 20 patients were in the omega-3 group, while 20 patients were in the placebo group (Figure 1).

Mean age in the case group (including 11 males and 9 females) was 37.7 ± 9.88 years and in the placebo group (including 10 males and 10 females) was 37.75 ± 9.52 years. There was no significant difference between the two groups in terms of age and gender.

In the first follow-up (after 3 months), the average number of ulcers, average duration of ulcers, site of ulcers, and USS of ulcer were significantly different between the two groups of omega-3 and placebo. But, there was no significant difference between the two groups in terms of the size of ulcer, mean pain intensity, and the ulcer-free period. In the second follow-up (after the 6 months), there was a significant statistical difference between the two groups in all characteristics of aphthous ulcers (Table 1). There was no statistically significant difference between all factors at the baseline value (Table 1).

Considering the fact that 7 out of 26 questions of the COMDQ questionnaire had a significant difference between the omega-3 and placebo groups at the initial examination, Mann–Whitney statistical analysis was used to compare the difference between the scores of the baseline questionnaire and the 3^rd^ month follow-up, as well as the difference between the score of the 3^rd^ month follow-up and the 6^th^ month follow-up between the omega-3 and placebo groups. The results of this analysis are presented in Table 2.

Intragroup comparisons with the Wilcoxon test revealed the differences in drug efficacy between the follow-up periods in each study group. It was found that most of the parameters in the omega-3 group were better than in the previous session; however, only the questions 2, 3, and 5 of the second part of the questionnaire were different in the placebo group (Table 3).

The mean score of the questionnaire in each group was obtained from all three occasions and showed a decrease in the mean score in the omega-3 group in the first and second follow-up. In the control group, by contrast, there was no decrease in the mean score (Table 4). By chi-square analysis and weighting mean, the mean score of the questionnaires in three sessions in two groups were compared. The difference between the groups in the three periods was significant (*P*=0.039). (Table 4).

Also, analysis of the results of the COMDQ questionnaire suggested that the consumption of spicy and sour foods increased the risk of discomfort in patients with RAS (OD: 9, 95% CI: 1.63, 49.44). In addition, toasted foods also caused discomfort in these patients (OD: 7.36, 95% CI: 1.33, 40.54). Also, the disease caused patients to underuse toothbrushes and dental floss (OD: 4.8, 95% CI: 1.2, 19.12). This disease also reduced social activities (OD: 6, 95% CI: 1.45, 24.68).

## 4. Discussion

In the present study, a reduction was observed in some clinical symptoms such as number of ulcers, duration of ulcers, and USS score in omega-3 patients at the end of the third month (first follow-up). In the second follow-up, all characteristics of the aphthous ulcer in the omega-3 group showed a significant improvement. The number, size, pain intensity, duration, site, and USS of the ulcer diminished while the ulcer-free period increased.

Only two studies have been carried out on the effects of omega-3 in the treatment of recurrent aphthous stomatitis reporting reduction of clinical effects, including the number, mean pain intensity, size, and the rate of recurrence of the ulcers [3, 28].

The USS criterion was used in this study to obtain the sum of the scores of the characteristics (size, number, duration, ulcer-free period, pain, and site). A decline was observed in mean USS after 3 and 6 months in the omega-3 group compared to the baseline. This criterion alone can be used as a standard tool for assessing the severity of aphthous stomatitis and for assessing the efficacy of treatments. This questionnaire is based on the patient's observations. The use of USS also enhances the reliability of clinical information to improve the integrity of disease history [27, 29].

Studies have argued that evidence of the anti-inflammatory effects of omega-3 fatty acid is dose-dependent and time-dependent, while the dose and time required to prevent or to treat the inflammation are still unclear [30] and require further studies on a larger sample size and different doses of omega-3. We used omega-3 1000 mg capsules in this study and performed three- and six-month follow-ups.

Some studies have reported beneficial effects of the systemic use of immunosuppressive drugs such as colchicine, prednisolone, levamisole, dapsone, and thalidomide in RAS patients due to the inflammatory and immunologic nature of the pathogenesis of RAS [31–34]. The goal of pharmacotherapy in RAS is to reduce the symptoms of the disease, but most medicines do not affect the frequency of disease. The drugs that can reduce the frequency include immunomodulatory agents such as omega-3, colchicine, prednisolone, and thalidomide. Colchicine, prednisolone, and thalidomide have many side effects [31–35], while omega-3 has the minimum side effects in this group of medicines and can be a supplement or in the form of food containing this fatty acid.

We also observed a significant improvement in the oral health-related quality of life in the omega-3 group compared to the placebo group after the 3-month and 6-month follow-ups based on the COMDQ questionnaire. This was consistent with the results of Elkhoulis study. They used the OHIP-14 questionnaire to assess the quality of life of RAS patients and reported improvement in their quality of life after 6 months [3]. The COMDQ questionnaire is a specific questionnaire for quality of life in patients with chronic mucosal disease including RAS [24]. Patients with RAS usually experience severe pain during talking, eating, drinking (especially hot and cold, spicy, and sour drinks), and swallowing. So, the digestion of the food is impaired. It also affects interpersonal communication problems, reduces self-esteem, and causes depression and isolation. All these suggest that the disease should affect the quality of life of patients [1, 3–5]. As a solution, the use of omega-3 leads to recovery of the oral function and social activities of patients and, in general, the improvement of the oral health-related quality of life.

Since RAS is a chronic mucosal inflammatory disease and inflammatory and immunological processes play a vital role in its pathogenesis, some studies have found a relationship between RAS and antibody-dependent lymphocytotoxicity, defect in subgroup cells of lymphocyte, and changes in the ratio of CD4 to CD8 lymphocytes [1, 6]. Omega-3 fatty acid causes variations in the expression of inflammatory elements through lipid mediators EPA and DHA. Indeed, EPA and DHA alter the cellular function of the polymorphonuclear leukocytes to regulate proliferation of the lymphocytes and specifically the activity and expression of the mRNA associated with the host antioxidant enzymes such as glutathione peroxide and superoxide dismutase. Therefore, they have an anti-inflammatory effect. EPA and DHA unsaturated fatty acids also prevent the arachidonic acid metabolism through the cyclooxygenase and lipooxygenase pathways, thus producing less inflammatory mediators (prostaglandins and cytokines). So, they prevent the tissue damage and help healing the ulcers [36–39].

## 5. Conclusion

During the last few years, only two trials have tried to evaluate the effect of omega-3 to recurrent aphthous ulcers. The results of the present study indicated that omega-3 supplements significantly improved the aphthous ulcer intensity and oral health-related quality of life in patients with recurrent aphthous stomatitis. It is recommended that future studies are performed with a larger sample size, a longer follow-up period, and with the use of different doses of omega-3 supplement to evaluate the effect of this medication.

## Figures and Tables

**Table 1:** Ulcer characteristic at baseline, after 3 months, and after 6 months.

Ulcer characteristic	Groups	Baseline		First follow-up after 3 months		Second follow-up after 6 months	
		Mean ± standard deviation	*P* value	Mean ± standard deviation	*P* value	Mean ± standard deviation	*P* value
Average size of ulcer	Placebo	4.75 ± 0.55	0.079	4.65 ± 0.48	0.082	4.6 ± 0.50	<0.0001^*∗*^
	Omega-3	5.11 ± 0.70		4.37 ± 0.48		3.5 ± 1.27	
Average number of ulcer	Placebo	2.65 ± 0.81	0.06	2.45 ± 0.75	0.018^*∗*^	2.45 ± 0.75	<0.0001^*∗*^
	Omega-3	3.6 ± 0.88		1.9 ± 0.64		1.35 ± 0.67	
Average duration of ulcer (in weeks)	Placebo	8.83 ± 1.47	0.389	8.83 ± 1.47	<0.0001^*∗*^	8.57 ± 1.51	<0.0001^*∗*^
	Omega-3	9.69 ± 0.63		4.94 ± 0.93		3.84 ± 2.03	
Ulcer free period (in weeks)	Placebo	3.05 ± 0.68	0.07	3.1 ± 0.71	0.657	3.1 ± 0.71	<0.005^*∗*^
	Omega-3	2.55 ± 0.60		3.2 ± 0.69		3.7 ± 1.94	
Pain	Placebo	7.3 ± 1.34	1	7.2 ± 1.39	0.075	7.2 ± 1.36	<0.0001^*∗*^
	Omega-3	8.15 ± 1.30		4.9 ± 0.96		3.65 ± 1.78	
Mucosal site	Placebo	4.15 ± 0.93	0.474	3.75 ± 0.85	<0.0001^*∗*^	3.75 ± 0.78	<0.0001^*∗*^
	Omega-3	4.4 ± 1.23		2.85 ± 2.3		2 ± 1.02	
USS^ꞷ^ (ulcer severity score)	Placebo	31.1 ± 2.61	0.08	30.35 ± 2.3	<0.0001^*∗*^	30.3 ± 2.29	<0.0001^*∗*^
	Omega-3	33.36 ± 2.02		25.75 ± 4.53		14.95 ± 6.25	

^*∗*^ *P* value is significant; ^ꞷ^ulcer severity score.

**Table 2:** Intergroup comparisons of the questions differences between the baseline-first follow-up and first follow-up-second follow-up.

Questions	Difference between baseline and first follow-up	Difference between first and second follow-ups
*Pain and functional limitation*		
(1) How much do certain types of food⁄drink cause you discomfort (spicy food, acidic food)?	<0.0001^*∗*^	<0.0001^*∗*^
(2) How much does your oral condition cause you to limit the types of food⁄drinks you consume?	<0.0001^*∗*^	<0.0001^*∗*^
(3) How much do certain food textures cause you discomfort (rough food, crusty food)?	<0.0001^*∗*^	<0.0001^*∗*^
(4) How much does your oral condition cause you to limit the textures of the food you consume?	<0.0001^*∗*^	<0.0001^*∗*^
(5) How much does the temperature of certain foods⁄drinks cause you discomfort?	<0.0001^*∗*^	<0.0001^*∗*^
(6) How much does your oral condition cause you to limit the temperature of the foods⁄drinks you consume?	<0.0001^*∗*^	0.002^*∗*^
(7) How much does your oral condition lead to discomfort when carrying out your daily oral hygiene routine (brushing, ﬂossing)?	<0.0001^*∗*^	0.001^*∗*^
(8) How much does your oral condition cause you to limit your daily oral hygiene routine (brushing, ﬂossing, mouthwash usage)?	<0.0001^*∗*^	0.002^*∗*^

*Medication and treatment (including mouthwashes, gels, creams, ointments, injections, tablets, and infusions)*		
(1) How much do you feel you need medication to help you with activities of daily life (talking and eating)?	<0.0001^*∗*^	>0.05
(2) How satisﬁed are you with the medication being used to treat your oral condition?	<0.0001^*∗*^	0.03^*∗*^
(3) How concerned are you about the possible side eﬀects of the medications used to treat your oral condition?	<0.0001^*∗*^	>0.05
(4) How much does it frustrate you that there is no single standard medication to be used in your oral condition?	<0.0001^*∗*^	0.009^*∗*^
(5) How much does the use of the medication limit you in your every day life (routine ⁄ the way you apply or take your medications)?	<0.0001^*∗*^	>0.05
(6) How much does it bother you that there is no cure for your oral condition?	<0.0001^*∗*^	0.014^*∗*^

*Social and emotional*		
(1) How much does your oral condition get you down?	<0.0001^*∗*^	<0.001^*∗*^
(2) How much does your oral condition cause you anxiety?	<0.0001^*∗*^	0.007^*∗*^
(3) How much does your oral condition cause you stress?	<0.0001^*∗*^	0.003^*∗*^
(4). How much does the unpredictability of your oral condition bother you?	<0.0001^*∗*^	<0.001^*∗*^
(5) How much does your oral condition cause you to worry about the future (spread of the condition, possible cancer risk)?	0.002^*∗*^	0.026^*∗*^
(6) How much does your oral condition make you pessimistic about the future?	<0.0001^*∗*^	>0.05
(7) How much does your oral condition disrupt social activities in your life (social gatherings, eating out parties)?	<0.0001^*∗*^	0.03^*∗*^

*Patient support*		
(1) How satisfactory do you consider the information available to you regarding your oral condition?	0.043^*∗*^	>0.05
(2) How satisfied are you with the level of support and understanding shown to you by family regarding this oral condition?	0.602	>0.05
(3) How satisfied are you with the level of support and understanding shown to you by friends⁄work colleagues regarding your oral condition?	1	>0.05
(4) How isolated do you feel as a result of this oral condition?	<0.0001^*∗*^	0.03^*∗*^

^*∗*^ value is significant.

**Table 3:** Intragroup comparisons of the questions differences between the baseline-first follow-up and first-second follow-ups.

Groups			
Omega-3		Placebo	
Difference between baseline-first follow-up	Difference between first-second follow-ups	Difference between baseline-first follow-up	Difference between first-second follow-ups
*Pain and functional limitation*			
(1) How much do certain types of food⁄drink cause you discomfort (spicy food, acidic food)?			
<0.0001^*∗*^	<0.0001^*∗*^	>0.05	>0.05
(2) How much does your oral condition cause you to limit the types of food⁄drinks you consume?			
<0.0001^*∗*^	<0.0001^*∗*^	>0.05	>0.05
(3) How much do certain food textures cause you discomfort (rough food, crusty food)?			
<0.0001^*∗*^	<0.0001^*∗*^	>0.05	>0.05
(4) How much does your oral condition cause you to limit the textures of the food you consume?			
<0.0001^*∗*^	<0.0001^*∗*^	>0.05	>0.05
(5) How much does the temperature of certain foods⁄drinks cause you discomfort?			
<0.0001^*∗*^	<0.0001^*∗*^	>0.05	>0.05
(6) How much does your oral condition cause you to limit the temperature of the foods⁄drinks you consume?			
<0.0001^*∗*^	<0.0001^*∗*^	>0.05	<0.05
(7) How much does your oral condition lead to discomfort when carrying out your daily oral hygiene routine (brushing, flossing)?			
0.012^*∗*^	0.012^*∗*^	>0.05	<0.05
(8) How much does your oral condition cause you to limit your daily oral hygiene routine (brushing, flossing, mouthwash usage)?			
<0.001^*∗*^	<0.001^*∗*^	>0.05	<0.05

*Medication and treatment (including mouthwashes, gels, creams, ointments, injections, tablets, and infusions)*			
(1) How much do you feel you need medication to help you with activities of daily life (talking and eating)?			
<0.001^*∗*^	<0.001^*∗*^	>0.05	<0.05
(2) How satisfied are you with the medication being used to treat your oral condition?			
<0.0001^*∗*^	<0.0001^*∗*^	<0.0001^*∗*^	<0.0001^*∗*^
(3) How concerned are you about the possible side effects of the medications used to treat your oral condition?			
0.003^*∗*^	0.003^*∗*^	<0.0001^*∗*^	<0.0001^*∗*^
(4) How much does it frustrate you that there is no single standard medication to be used in your oral condition?			
>0.05	>0.05	>0.05	>0.05
(5) How much does the use of the medication limit you in your everyday life (routine⁄the way you apply or take your medications)?			
>0.05	>0.05	0.012^*∗*^	0.013^*∗*^
(6) How much does it bother you that there is no cure for your oral condition?			
<0.005^*∗*^	<0.005^*∗*^	>0.05	>0.05

*Social and emotional*			
(1) How much does your oral condition get you down?			
0.026^*∗*^	0.016^*∗*^	>0.05	>0.05
(2) How much does your oral condition cause you anxiety?			
0.004^*∗*^	0.004^*∗*^	>0.05	>0.05
(3) How much does your oral condition cause you stress?			
0.002^*∗*^	0.003^*∗*^	>0.05	>0.05
(4) How much does the unpredictability of your oral condition bother you?			
0.007^*∗*^	0.008^*∗*^	>0.05	>0.05
(5) How much does your oral condition cause you to worry about the future (spread of the condition, possible cancer risk)?			
>0.05	>0.05	>0.05	>0.05
(6) How much does your oral condition make you pessimistic about the future?			
0.046^*∗*^	0.026^*∗*^	>0.05	>0.05
(7) How much does your oral condition disrupt social activities in your life (social gatherings, eating out parties)?			
0.024^*∗*^	0.023^*∗*^	>0.05	>0.05

*Patient support*			
(1) How satisfactory do you consider the information available to you regarding your oral condition?			
>0.05	>0.05	>0.05	>0.05
(2) How satisfied are you with the level of support and understanding shown to you by family regarding this oral condition?			
>0.05	>0.05	>0.05	>0.05
(3) How satisfied are you with the level of support and understanding shown to you by friends⁄work colleagues regarding your oral condition?			
>0.05	>0.05	>0.05	>0.05
(4) How isolated do you feel as a result of this oral condition?			
>0.05	>0.05	>0.05	>0.05

^*∗*^ *P* value is significant.

**Table 4:** Mean of the sum question's score at baseline, after 3 months, and after 6 months.

Groups	Mean at baseline	Mean after 3 months	Mean after 6 months	
Control	83.33	87.29	87.54	*P*=0.039^*∗*^
	*P*=0.2			
Omega-3	89.58	56.95	44.45	
	*P* < 0.001^*∗*^			

^*∗*^ *P* value is significant.

## Data Availability

The data used (questionnaire of ulcer severity score and the chronic oral mucosal disease questionnaire) to support the findings of this study are available from the corresponding author upon request.
